# Should the Human Microbiome Be Considered When Developing Vaccines?

**DOI:** 10.1371/journal.ppat.1001190

**Published:** 2010-11-18

**Authors:** Rosana B. R. Ferreira, L. Caetano M. Antunes, B. Brett Finlay

**Affiliations:** Michael Smith Laboratories, University of British Columbia, Vancouver, British Columbia, Canada; The Fox Chase Cancer Center, United States of America

The human microbiome, especially in the intestinal tract has received increased attention in the past few years due to its importance in numerous biological processes. Recent advances in DNA sequencing technology and analysis now allow us to better determine global differences in the composition of the gut microbial population, and ask questions about its role in health and disease. Thus far, roles of these commensal bacteria on nutrient acquisition, vitamin production, and intestinal development have been identified [1]. Furthermore, resistance or susceptibility to a number of diseases, including inflammatory bowel disease, obesity, enteric infections, and most recently ectopic diseases, have been linked to the intestinal microbiota [1], [2]. Data on the mechanisms through which the intestinal microbiota impacts host immune development have also begun to emerge [2]. The impact of the intestinal microbiota on host physiology is undeniable, and experiments using germ-free, mono-, and poly-colonized mice have addressed many aspects of the microbiota's influence on the mammalian immune system.

Despite all the increased attention on the interface between the microbiota and host immune responses, it is still unclear whether these commensal bacteria affect the efficacy of vaccines. Due to its impact in the development of immune function, both in the gut and other organs, it is reasonable to consider that the intestinal microbiota will significantly affect how individuals respond to vaccine antigens [3], [4]. For example, segmented filamentous bacteria present in the intestinal microbiota have been shown to induce maturation of intestinal T cell adaptive functions [5]. Moreover, it has been shown that the intestinal microbiota exerts a profound effect on the metabolism of certain drugs and toxins [1], [6], and this may also indicate that oral vaccines could be differentially processed by the body depending on variations in microbial communities between individuals. Hence, the microbiota could be an underappreciated yet important player to consider in the development of vaccines, and also may help explain some of the discrepancies observed in vaccine efficacy in different populations around the world.

Clinical trials testing the efficacy of oral vaccines against polio, rotavirus, and cholera have showed a lower immunogenicity of these vaccines in individuals from developing countries when compared to individuals from the developed world [7]–[11]. Clinical trials for a killed oral cholera vaccine in Swedish and Nicaraguan children have also shown blunted antibody responses in Nicaraguan children compared to Swedish children [11]. In a study testing a live cholera oral vaccine, Lagos and colleagues [12] demonstrated that excessive bacterial growth in the small intestine of children in less developed countries might contribute to the low antibody response to the vaccine. Different vaccine strains of *Shigella flexneri* also showed differential protection on individuals from developing countries. In a study testing Bangladeshi adults and children, no significant immune response to this vaccine was mounted, although the same antigen was reactogenic in North American individuals [13]. Altogether, these data highlight that individuals from different parts of the world can mount different immune responses to the same vaccine. Several hypotheses that may explain this phenomenon exist. For instance, socioeconomic conditions, nutritional status, host genetics, and earlier exposure to related microorganisms are some of the aspects that could contribute to the disparity in the vaccine efficacies in different populations. However, one poorly explored possibility is that the composition of the intestinal microbiota of these individuals may also be a determining factor of vaccine efficacy. In a way analogous to the hygiene hypothesis [14], which states that reduced exposure to microorganisms at an early age may lead to increased susceptibility to allergies, it is possible that the gut microbiota of individuals with increased exposure to microorganisms (and therefore antigens) make them more tolerant to vaccination, being unable to mount a proper response compared to individuals living in better socioeconomic conditions.

Discerning the effects of genetic and environmental factors on vaccine efficacy is a challenging task. Large clinical trials involving individuals from different areas of the world will likely be required to shed light on whether the blunt immune responses to some of the oral vaccines mentioned herein are a consequence of genetic factors or environmental variations, such as the gut microbial community. Studies involving immigrant volunteers could be useful in addressing this issue by providing a clear distinction between the effects of genetics and the environment. Although this is still an open question, data in the literature suggest a more direct link between the intestinal microbiota composition and the development of immune responses to certain vaccine antigens. For instance, the use of antibiotics in chickens has been shown to increase the antibody response following immunization [15]. Because antibiotic treatment will have profound effects on the intestinal microbiota, it is tempting to hypothesize that the microbial populations of these animals are important players in their immunological response to the vaccine antigens. Furthermore, certain probiotic strains have been shown to enhance antibody responses to oral vaccines against rotavirus [16], *Salmonella* [17], polio [18], and cholera [19] in human volunteers, and this effect was observed after a short period (1–5 weeks) of probiotic treatment. The positive effect of probiotics on immune responses was also seen in parenterally administered vaccines against diphtheria, tetanus, *Haemophilus influenzae* type B, and hepatitis B [20]–[22] in infants after a 6-month period. Because of the number of licensed oral-administered human vaccines available is limited, studies on how the intestinal microbiota affect parenterally administered human vaccines would have a more significant impact on human health. However, in all studies cited above, there was no long-term follow-up on the enhanced effects of the probiotics on vaccine efficacy. Additionally, more detailed studies on the establishment of the probiotic strains within the resident microbiota will be required to establish minimal doses and treatment regimens, important aspects that need to be addressed if the microbiota is to be considered in vaccine development in the future. It has also been suggested that prebiotics, which are compounds that can enhance the proliferation of certain commensals, can enhance the efficacy of oral vaccines. Recently, a well-studied fructo-oligosaccharide prebiotic has been shown to improve the efficacy of a vaccine against *Salmonella* infection [23]. In this study, administration of the prebiotic prior to vaccination improved host responses and rates of protection against infection in mice. Unfortunately, the authors were unable to show significant changes in microbiota composition, possibly due to the lack of detailed analyses. In another study, Vos et al. [24] showed that a prebiotic mixture containing galacto- and fructo-oligosaccharides enhanced systemic adaptive immune responses in a murine influenza vaccination model. In this case, increased proportions of certain members of the microbiota could be observed, suggesting a role for the microbial community in the increased host immune response.

Although some studies indicate that the microbiota may play an important role in vaccine efficacy, this area of research is still in its infancy. For instance, the mechanisms involved in the pro- and prebiotic enhancement of vaccine efficacy mentioned above are largely unknown. Nevertheless, current knowledge of the effect of the intestinal microbiota on the development of not only local but also systemic immune functions provides a direct link between commensal populations in the intestine and immune responses to vaccine antigens [3], [4]. We now have the tools to study and take advantage of what the microbiota has to offer in order to enhance host responses to vaccines, with the use of probiotics or prebiotics as adjuvants. Studies using animal models with defined intestinal microbial communities can be helpful to evaluate the effect of intestinal commensals on the immune response to vaccines. However, animal models can only partially elucidate this issue and, although cumbersome, studies in human volunteers will be essential in defining the effect of commensals in vaccine efficacy. We suggest that the study of the relationships between individual commensal populations of humans and responses to vaccines will be instrumental in our quest to improve general vaccine development. By taking into consideration the microbial populations of geographically diverse groups of individuals, we may be able to develop better-targeted vaccines that will improve protection against multiple pathogens.
